# An ERP Study on Decisions between Attractive Females and Money

**DOI:** 10.1371/journal.pone.0045945

**Published:** 2012-10-15

**Authors:** Jianmin Zeng, Yujiao Wang, Qinglin Zhang

**Affiliations:** 1 School of Psychology, Southwest University, Chongqing, China; 2 Key Laboratory of Cognition and Personality, Ministry of Education, China; University College London, United Kingdom

## Abstract

To investigate the neural processes of decision-makings between attractive females and money, we recorded 18 male participants' brain event-related potentials (ERPs) when they performed a novel task of deciding between viewing an attractive female's fuzzy picture in clear and gaining a certain amount of money. Two types of attractive females were included: sexy females and beautiful females. Several new electrophysiological discoveries were obtained as following. First, the beautiful females vs. money task (task B) elicited a larger positive ERP deflection (P2) than the sexy females vs. money task (task S) between 290 and 340 ms, and this probably related to the perception matching process between a visual input and an internal representation or expectation. Second, task S evoked greater negative ERP waves (N2) than task B during the time window of 340–390 ms, and this might relate to response conflict and cognitive monitoring for impulsive tendency. Third, the ERP positivity in task S was larger than task B in the time interval of 550–1000 ms, reflecting that sexy female images may have higher decision value for males than beautiful female images. Fourth, compared with choosing to gain money, choosing to view an attractive female evoked a larger late positive component (LPC) during the same time window, possibly because attractive females are more direct and evolutionarily earlier rewards for males than money amounts.

## Introduction

According to studies in economics and psychology [1], the term of reward contains at least three core characteristics. First, rewards are discounted as a function of time delay. Second, based on an equivalence function, different types of rewards can be traded with each other. Third, rewards reinforce work as incentives.

Among many kinds of rewards, money is an important reward in humans' social life and plays a fundamental role in the activity of economic exchange. In studies of monetary rewards, a person would carry out actions only when the benefits of one's actions outweighed their costs, according to economical theories [2].

In recent years, a lot of data have been accumulated relating to the human brain's sensitivity to monetary rewards [3]–[5]. These neuroimaging studies implicated that the activation of ventral striatum, medial prefrontal cortex, posterior cingulate cortex, lateral prefrontal and posterior parietal cortex were involved in monetary reward decision-makings. With respect to the event-related brain potential (ERP) studies investigating the brain processes involving monetary rewards, most of the experiments employed gamble tasks or risk tasks in which subjects chose between two options which may vary in outcome valence (gain or loss), outcome probability (such as 50% gain or loss) and outcome magnitude (large or small) [6]–[9]. For example, a study revealed that, anticipation of high-consequence outcomes triggered a greater positive potential than those involving low-consequence outcomes at around 150 ms [6].

People are often in contact with other individuals in order to adapt the complex natural and social environments. Behavioural evidence suggested that viewing the opposite sex (e.g., attractive females) can be regarded as a reward similar to other rewards such as food, drink or money, and had the same characteristics with them, meaning that the value of viewing the opposite sex was discounted by delay of viewing time, substituted for monetary rewards and reinforced work [1]. Therefore, decision-makings involving rewards of looking at the opposite sex probably obey the same set of economic principles in monetary rewards. An individual would engage in a certain social behavior (e.g., viewing opposite sex) only when the benefits can outweigh the costs of doing so, according to the social exchange theory [10].

Concerning the human brain's sensitivity to these rewards, a recent fMRI experiment revealed a dissociation between erotic pictures and monetary gains in the orbitofrontal cortex (though these rewards shared some common brain regions) in males [11]. Its posterior region (the posterior lateral OFC) was specifically stimulated by more basic erotic images (a primary reward), while its anterior region (the anterior lateral OFC, which is a more recent structure in human) was activated by monetary gains (a secondary reward). This finding indicated that the more abstract and complex the reward, the more its representation stimulated the anterior regions of the orbitofrontal cortex.

In addition, an ERP study showed that erotic pictures evoked significantly higher positive slow waves (PSW) than non-erotic stimuli between 500 and 750 ms [12]. Furthermore, results from previous ERP studies relating to the detection and analysis of emotional arousing picture stimuli showed that, emotional faces evoked a late positive potentials (LPC) compared with neutral stimuli [13]–[14]. Their findings demonstrated that the LPC was related to greater affective arousal and adaptive controls in intentional behaviours [13]. This was possibly because that emotional stimuli might have greater adaptive significance for the survival of human beings, and so prior and more salient processing in the human brain [14].

The above studies provided abundant data about the human brain responses to the money and attractive female rewards, respectively. To our knowledge, however, the neural process of human decision-making between viewing attractive females and gaining money was poorly understood, and the time course of the process can not be studied with precision using fMRI. Understanding these processes represents a better understanding of human decision-making between primary and secondary rewards. Referring to the experimental method, with high temporal resolution, event-related brain potentials (ERPs) technique can be time-locked to the onset of the presentation of external stimuli, and may provide a better means of evaluating cognitive processes prior to a response. Therefore, ERP-measurement would allow for more precise statements about the time course of the decision-making, thus may better suit for the purpose of monitoring early-phase of the process than neural imaging technique.

Here, we used ERPs together with a novel decision-making task to demonstrate the neural processing concerning the decision-making between attractive females and money. In the formal experiment of the present study, male participants chose between viewing an attractive female's fuzzy picture in clear for 1.5 second and gaining a certain amount of money (0.5–2 *fen*). The purpose of the present study was to investigate the spatiotemporal patterns of brain activity during the performance of decision-making tasks. First, we wanted to examine whether choosing to view an attractive female and choosing to gain money elicited different ERPs. Based on previous findings [12]–[13], we predicted that different decisions could be preceded by different ERPs. Second, we hypothesized that the decision-making in a sexy female vs. money task would evoke different ERPs, compared with a beautiful female vs. money task, according to previous study [12]. To the best of our knowledge, this may be the first ERP study investigating the electrophysiological correlates of decision-making between attractive females and money.

## Materials and Methods

### Participants

Eighteen healthy heterosexual male undergraduates participated in the formal part of this experiment (mean age, 21.1 years; age range, 19–23 years). The reason that we recruited only male participants was that men, who were not influenced by menstrual cycle effects [12], are supposed to be easily attracted by attractive females and their responses to visual sexual stimuli are generally more obvious than women, both in behavioral performance and physical arousal [11], [15]. All subjects were right-handed and had normal or corrected-to-normal vision. They reported no history of current or past psychiatric or neurological illness. These subjects all signed written informed consent prior to participation and were paid after the experiment. This experiment was approved by the Administration Committee of Psychological Research in Southwest University.

### Stimuli and procedure

There were 2 pilot experiments and 1 formal experiment in this study. In order to increase the homogeneity of the experimental material in our study, in the first pilot experiment, 31 male undergraduates rated 1800 sexy female pictures and 1800 beautiful female pictures separately. All pictures were selected beforehand by experimenters from the Internet according to the following criteria: each picture contained only a single Asian female with a clearly visible face in beautiful females and a clearly body outline in sexy females; beautiful females should dress ordinarily and their faces looked beautiful and their facial expression presented positive or pleasant, while sexy females should dress bikini or underwear and their postures looked sexy. Photos which were blurry or small, or in which females were back to us, emotionally negative, or appeared younger than 18 years old were eliminated. Before the first pilot experiment, male subjects were given a gentle reminder that it was normal to enjoy attractive female pictures for males and they needed not feel shameful for this. These raters did not participant in subsequent experiments. The 2400 higher-rate pictures (1200 beautiful and 1200 sexy, respectively) were picked out and underwent a fuzzy operation (11% pixels of the original pictures) for the second pilot experiment and the formal experiment.

The second pilot experiment had two aims. First, for each participant, to divide beautiful and sexy female pictures respectively into high-attractiveness and low-attractiveness sets. Second, to explore the appropriate range of money amounts to be used in the formal experiment. Accordingly, it contained two phases. In the first phase, in each trial, each of 76 male subjects (not containing subjects in the 1^st^ pilot experiment) faced an attractive female's fuzzy picture and answered the question “How much would you like to see this fuzzy picture in clear? High or Low?" Each participant was required to keep the percentage of each category (high or low) between 45% and 55%, so that he would divide the beautiful and sexy female pictures respectively into two sets with roughly equal numbers of pictures. They had a random chance of one out of ten to view the clear version of attractive female pictures if they chose “High", but no chance if “Low". This could encourage subjects to express their real desire. In this way, we established individualized pictures of four kinds (High or Low by sexy or beautiful) for each subject. After the above mentioned three filtering procedures (the experimenters' selecting, the 31 subjects' rating on the clear pictures and the 76 subjects' rating on the fuzzy pictures), we assumed that female pictures within a category (e.g., High sexy) for a subject should have similar attractiveness for this subject. In the second phase, in each trial, each subject made a decision whether to view a clear version of an attractive female's fuzzy picture (all from his “High" pictures) or to obtain a certain amount of money (randomly varying within 0–5 *fen* with increment of 0.2 *fen*; 1 *US penny*≈6.4 *fen*), wherein money was presented with a coin picture which had undergone the same fuzzy operation as the attractive female pictures. For each kind of pictures (“High" sexy and beautiful female pictures), we estimated with logistic statistics a cash equivalent (CE) for each subject, between which and the corresponding pictures the subject would choose with equal possibility or they were indifferent between them. These CEs were used to decide the range of the amounts of money used in the formal experiment. In the logistic statistics, we used the following model: 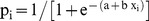, where p_i_ was the probability of a subject's special response (choosing an attractive female or choosing a certain amount of money) in the i^th^ case, x_i_ was the money amount in the i^th^ case, and a and b were indexes needed to be solved out by fitting this model to the actual data. Utilizing that the above formula implies p_i_ = 0.5 if and only if x_i_ = −a/b, we calculated the CE as −a/b. According to the above formulae, for the pictures included in this regression, if we had fixed the money amount as this CE, then the subject would have chosen the pictures and the CE with a probability of 50% respectively.

18 out of 76 subjects in the second pilot experiment volunteered to further participate in the formal experiment. The materials of the formal experiment contained two kinds of attractive female pictures: Low sexy female pictures and High beautiful female pictures (this made two kinds of pictures be more comparable so as to increase the possibility of finding the common trend in two conditions), both of which had not been viewed in clear by subjects in the previous 2 pilot experiments (to avoid habituation effect). Again, each picture had two versions: one was fuzzy and the other was clear.

The formal experiment included two tasks: the first task S (sexy female vs. money) and the second task B (beautiful female vs. money). We adopted a block design rather than blending two kinds of tasks together, because the fixed money amounts differed in task S and L and did not appear in the decision screen in the formal part of the formal experiment. If we blended two kinds of tasks together, subjects needed to firstly figure out what was the optional money amount for the current picture in each trial. This would complicate subjects' decision processes.

Procedures for both tasks were identical to each other. Therefore, we explain only task S here, which included two phases. Firstly, subjects carried out the decisions (between attractive females and money) again. The procedure was similar to the second phase of the second pilot experiment. A main difference was that the money amount was adjusted to 0–3 *fen* (accurate to two decimal places) with an increment of 3/49 *fen*, because the second pilot experiment had shown that the CEs of subjects generally varied from 0.5 to 2 *fen*. Based on each subject's choices, the procedure calculated his formal-experiment CE (CE hereafter) for this kind of pictures with a simple algorithm. We did not record ERPs in this phase.

**Figure 1:**
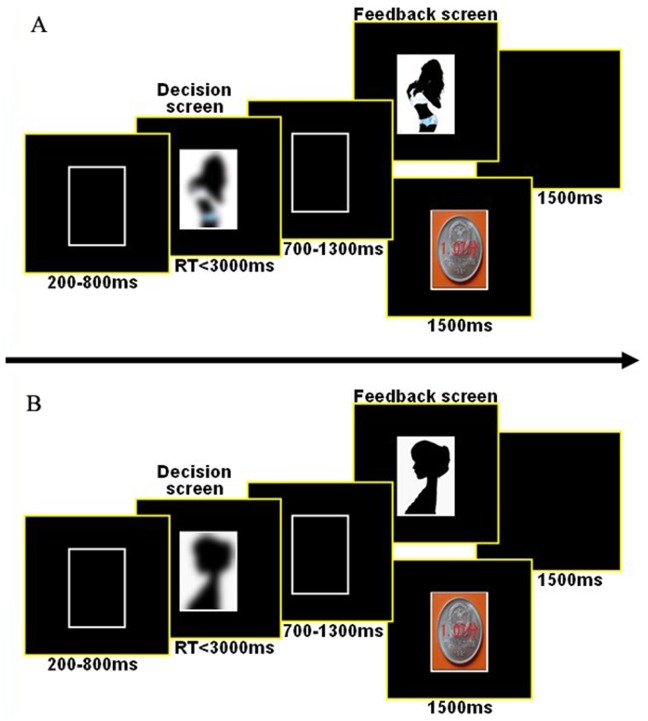
Sequence of stimuli in a typical trial in the second phase of the formal experiment. Times under boxes represent stimulus duration. Indicative instead of original images of attractive females were used here for a demonstration purpose. (A) Task S (decision-making between viewing a *sexy* female in clear and gaining money); (B) Task B (decision-making between viewing a *beautiful* female in clear and gaining money).

In the second phase, at the beginning, each subject was instructed that he needed to choose between a fixed amount of money (actually his CE but this was not told him) and each of many pictures; he needed to beforehand remember the money amount because it would no longer appear in the decision screens hereafter. At the beginning of each trial, a rectangle with white border appeared in the center of the screen for 200–800 ms in order to make subjects pay attention to the task, followed by a fuzzy picture of an attractive female in it, without money appearing. Participants pressed the corresponding keys within 3000 ms to indicate whether to view a clear version of the attractive female's fuzzy appearance or to get the money (CE). The rectangle remained in the screen for another 700–1300 ms after his decision but the fuzzy picture disappeared. The feedback (a clear picture of the attractive female or money according to subject's decision) displayed in the rectangle for 1500 ms. If the subject did not make a decision during this trial, then he would get the reminding “You didn't make a decision" instead of an attractive female or money. The inter-trial interval lasted for 1500 ms as a blank screen (Figure 1).

This second phase consisted of 200 trials, sequentially including 20 practical trials, 80 formal trials, a break, 20 practical trials, and 80 formal trials. ERPs were recorded for the 160 formal trials in this phase. The key pressing was counter-balanced within and between subjects: half subjects pressed “f" for choosing attractive female images and “j" for choosing money in the first 100 trials, and did the converse in the remaining 100 trials; the other half subjects had the opposite arrangement.

The participants were seated in a quiet room facing a computer screen placed about 80 cm away from their eyes. All stimuli were presented against the black background and were positioned in the center of the computer screen. Participants were asked to avoid eye blinking or any body movement and to keep their eyes fixated on the centre of the screen while performing the task. After they completed the experiment, the subjects got the real decision payoffs according to their choices in addition to their participating payoffs and they had known this point at the beginning of this formal experiment.

### Electrophysiological recording and analysis

An elastic cap with electrodes of 64 scalp sites was used to record subjects' brain electrical activity (Brain Product, Munchen, Germany), with the references placed on the left mastoid. The vertical electrooculogram (VEOG) generated from blinks and vertical eye movements was also recorded by using miniature electrodes placed approximately 1 cm above and below the subject's right eye, and the horizontal electrooculogram (HEOG) by the right side of the right eye and the left side of the left eye. All electrode impedances were maintained below 10 kΩ. The EEG and EOG signals were amplified and digitized with a sampling rate of 500 Hz and a bandpass of 0.1–100 Hz. The EEGs went through the following steps of offline preprocessing. They were rereferenced to an averaged mastoid reference. Eye movement artifacts (eye blinks and movements) were corrected. The EEGs was then filtered with a high cutoff of 16 Hz, 12 dB/oct. They were then segmented and baseline-corrected. Segments whose peak voltages exceeded ±80 µV after correction were excluded before averaging. All these steps were performed with the Brain Vision Analyser software (Brain Products).

**Figure 2:**
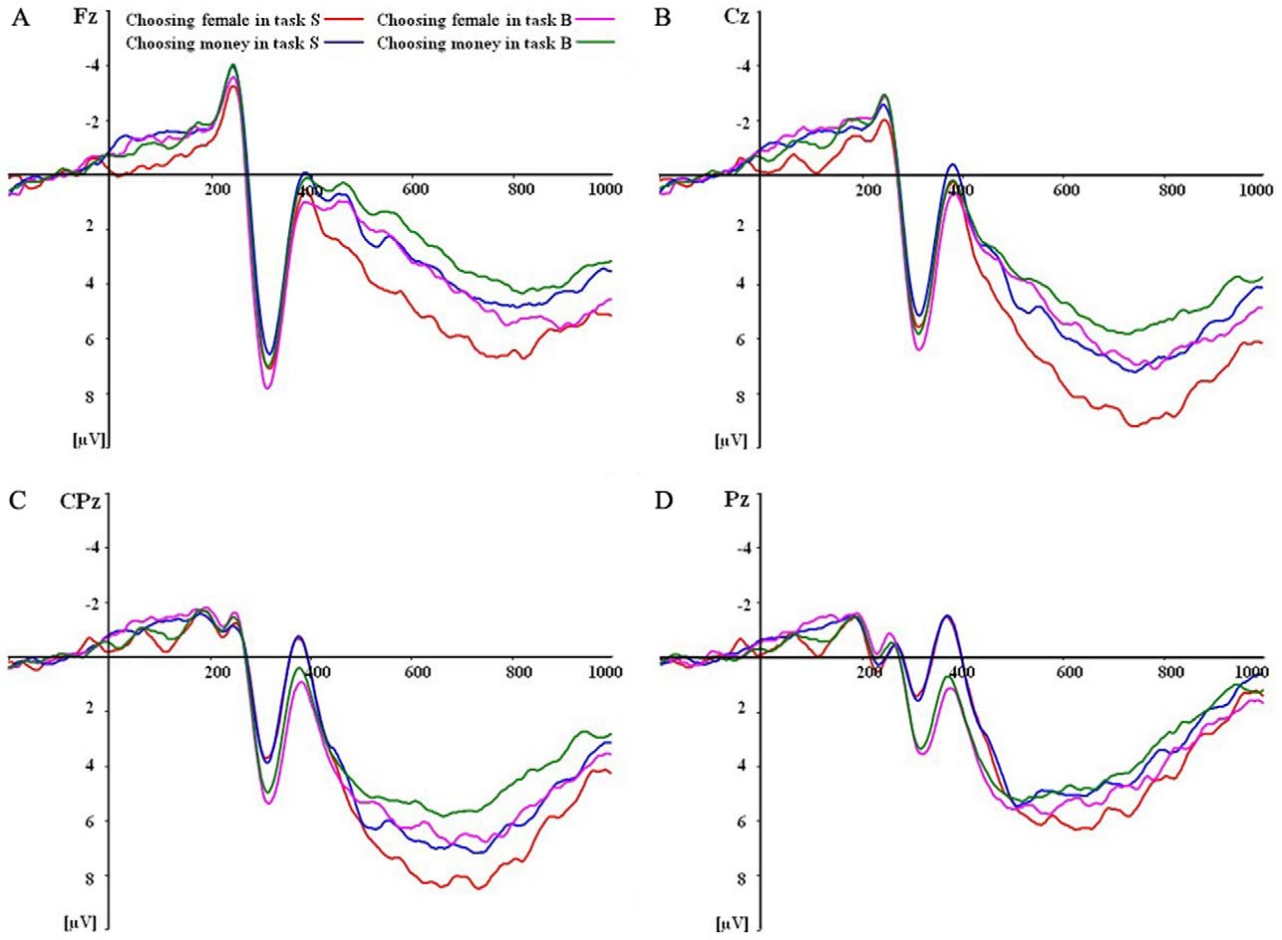
Grand average ERPs in the decision-making phase. Grand average ERPs of the four conditions at Fz, Cz, CPz and Pz electrode points when subjects made decisions.

**Figure 3:**
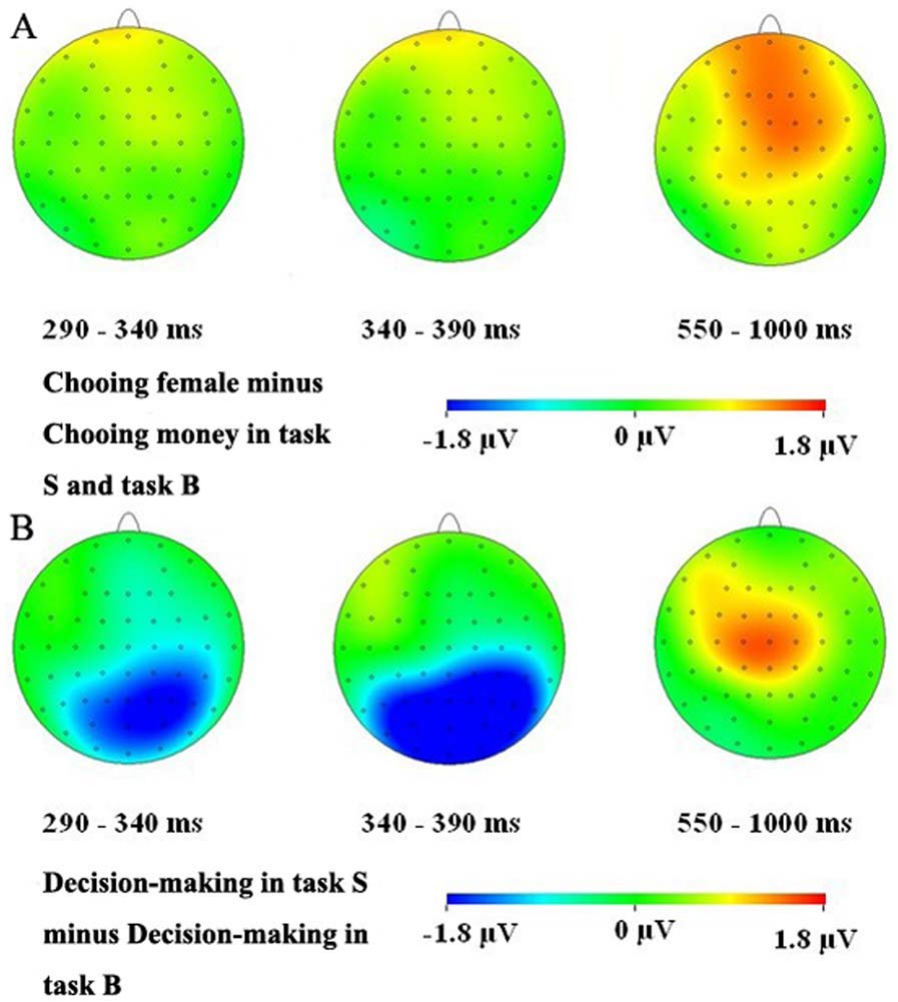
Topographic maps of voltage amplitude difference of two kinds of decisions and tasks. (A) choosing attractive females minus choosing money in task S and B in three time windows of 290–340 ms, 340–390 ms and 550–1000 ms, respectively. (B) decision-making in task S minus that in task B in three time windows of 290–340 ms, 340–390 ms and 550–1000 ms, respectively.

The ERP waveforms were time-locked at the onset of stimuli appearance in the decision screen. The averaged time interval for ERP was 1200 ms including 1000 ms post-stimulus waveform and 200 ms pre-stimulus baseline. On the basis of the grand averaged ERPs and topographical maps (see Figs. 2 and 3), the following 9 electrode points were chosen for statistical analysis in the time window of 290–340 ms: CP2, CP4, P1, P2, P4, P6, Pz, PO4, POz; 15 electrode points were chosen for statistical analysis in the time window of 340–390 ms: CP2, CP4, P1, P2, P3, P4, P6, Pz, PO3, PO4, PO8, POz, O1, O2, Oz, and 22 electrode points were chosen for statistical analysis in time window of 550–1000 ms: AF3, AF4, C1, C2, C3, C4, CP1, CP2, C3, CP4, CPz, Cz, F1, F2, F3, F4, FC1, FC2, FC3, FC4, FCz, and Fz. Mean amplitudes in each time window were respectively analyzed using three-way repeated-measures analysis of variance (ANOVA) with the Greenhouse-Geisser correction applied to p-values for deviations.

## Results

### Behavioral results

During the decision-making phase, we defined the condition of decision-making between sexy females and money as task S, and that between beautiful females and money as task B. The results of a 2 (task type: task S or task B)×2 (decision type: choosing an attractive female or choosing money) repeated-measures ANOVA of reaction time revealed significant main effects of both task type [*F*(1, 17) = 22.008, *p* = 0.000] and decision type [*F*(1, 17) = 12.900, *p* = 0.002], but their interaction was not significant [*F*(1, 17) = 0.600, *p* = 0.449]. The mean reaction time (RT) in task S was significantly longer than in task B, and the mean reaction time of choosing an attractive female was significantly longer than that of choosing money. Specifically, the mean reaction time of choosing money in Beautiful female vs. money condition (721.01 ms) was the fastest, and the subjects were slowest to respond in choosing sexy females (886.91 ms). The reaction time for choosing beautiful females and choosing money in Sexy female vs. money condition fell between the two, with the mean reaction times of 782.73 ms and 809.06 ms respectively.

### Electrophysiological scalp data

ERP waveforms evoked by four conditions after the onset of stimuli in the decision screen are shown in Figure 2: choosing attractive females in task S, choosing money in task S, choosing attractive females in task B, and choosing money in task B. The P2, N2, and late positive components (LPC) in 550–1000 ms time window were elicited by all the four conditions. From ERP waveforms and the voltage amplitude difference (see Figs. 2 and 3), we found that the decision-making in task B elicited a more positive ERP deflection (P2) than that in task S during the time window of 290–340 ms after onset of the stimuli. Subsequently, task S elicited a more negative ERP deflection (N2) than task B in the time interval between 340 and 390 ms. Moreover, in the time interval of 550–1000 ms, decisions elicited a more positive ERP deflection in task S than task B, and choosing-an-attractive-female decision elicited a more positive ERP deflection than choosing-money decision. Therefore, mean amplitudes in the time windows of 290–340 ms, 340–390 ms and 550–1000 ms were respectively analyzed using three-way repeated-measures ANOVAs.

A 2 (task type: task S or task B)×2 (decision type: choosing an attractive female or choosing money)×9 (electrodes) repeated measures ANOVA was conducted in the time window of 290–340 ms. Results revealed that there was a main effect of task type, *F*(1, 17) = 15.217, *p* = 0.001, while the main effect of decision type was not significant, *F*(1, 17) = 0.210, *p* = 0.653, and the interaction of task type×decision type was not significant, *F*(1, 17) = 0.277, *p* = 0.606. Mean amplitude was more positive for task B than for task S in 290–340 ms (see Fig. 3). The results of a 2 (task type: task S or task B)×2 (decision type: choosing an attractive female or choosing money)×15 (electrodes) repeated measures ANOVA showed that there was also a main effect of task type in the time window of 340–390 ms, *F*(1, 17) = 32.111, *p* = 0.000, and the main effect of decision type was not significant, *F*(1, 17) = 1.180, *p* = 0.292. Mean amplitude was more negative for task S than for task B in 340–390 ms (see Fig. 3). Likewise, the interaction of task type×decision type was not significant, *F*(1, 17) = 0.542, *p* = 0.471. Subsequently, a 2 (task type: task S or task B)×2 (decision type: choosing an attractive female or choosing money)×22 (electrodes) repeated-measures ANOVA showed that the main effect of task type and decision type were both significant in the time window of 550–1000 ms, *F*(1, 17) = 5.134, *p* = 0.037, *F*(1, 17) = 8.818, *p* = 0.009. Mean amplitude was more positive for choosing an attractive female than for choosing money, and mean amplitude was more positive for task S than task B (see Fig. 3). Also, there was no significant interaction between task type and decision type, *F*(1, 17) = 0.702, *p* = 0.414. Finally, the main effects of electrode site were significant or marginally significant in all these time windows; there were no two-way interactions between electrode site and the other two factors; there was no three-way interaction among all factors. These electrophysiological results demonstrated that task S and task B or choosing attractive females and choosing money might relate to the very same neural substrates rather than involve distinct neural substrates, given that they all elicited an LPC but were distinguished only by amplitude and there were no interactions involving electrode site.

## Discussion

The main goals of the present study were to verify whether the decision-making processes of choosing attractive females and choosing money have differential neurophysiological mechanisms and, if so, to identify different processing stages in the decision-making between attractive female rewards and money rewards. In order to realize these goals, we devised a novel task of decision between viewing an attractive female clearly and gaining a certain amount of money for healthy heterosexual male volunteers and recorded their brain EEG responses. Our experimental paradigm and procedure were elaborately designed so that male subjects would truly express their evaluation and decisions. Using this framework, we obtained several discoveries. First, the response time (RT) of choosing to view attractive females was significantly longer than choosing to gain money. The reason might be that choosing to gain money represented an optimal action (if a participant chose money, 1 *fen* could afford him to use Internet to browse attractive females for 1.5 minute; if he chose an attractive female, he could view the attractive female for only 1.5 second), and so a subject generally hesitated more before he chose to view an attractive female in clear than to gain money. Second, the type of attractive female pictures influenced the response time of decision-making in males. Specifically, behavioral results indicated that the RT for task S was longer than task B. The reason might be that the information of sexy females captured a male's attention easier and hindered their performance (delayed reaction time) in the decision-making task. These results were consistent with the notion of evolutionary theory that the erotic signal can easily attract males' attention, and this played an important role in mating and reproducing decisions for human beings [16]. Another possible reason was, the CEs for sexy female images were generally larger than those for beautiful ones, and so both options in task S (sexy images and higher money amount) were more attractive than those in task B (beautiful images and lower money amount), and so subjects would feel more conflicted in task S, which lengthened the reaction time in task S.

Scalp ERP analysis yielded several new interesting findings: the positivity in the time interval of 290–340 ms (P2) in task B was significantly larger than task S. Moreover, in the time window of 340–390 ms, the decision-making in task S elicited a greater negative component (N2) than in task B. Finally, the decision-making in task S elicited a greater late positive component (LPC) than in task B during the 550–1000 ms time window, and choosing an attractive female evoked a greater LPC than choosing money. We discuss the implications of these findings in terms of the association between ERP components and decision-making as follows.

The decision-making in task B elicited a more positive ERP deflection (P2) than in task S following onset of an attractive female stimulus during 290–340 ms. The P2 has been often studied in visual search [17], language processes [18], and memory recognition tasks [19]. Multiple evidence demonstrated that the P2 as a part of cognitive matching system, might reflect general neural processes that compared an external visual input with an internal memorial information [17], [19]–[20]. In the present study, beautiful females were characterized with their attractive faces, while sexy females were featured by their erotic postures, and participants might focus on females' faces and postures during task B and task S respectively. Due to the complexity and specialization of human brains processing human faces, the beautiful females may require more cognitive resources in perceptual-matching process than sexy females, thus elicited larger P2. This result could not be explained by sexy arousal, because if this was the case, the sexy females would have elicited larger P2, opposite to the actual result. This result indicated that during the early processing of the decision-making, the perception process may be different for beautiful and sexy female tasks.

The decision-making in task S demonstrated a greater negativity (N2) at 340–390 ms than in task B. Previous work indicated that the N2, which was regarded as a component of mismatch negativity or mismatch detector [21], often appeared in go/no-go tasks [22] and reflected a kind of cognitive inhibitory mechanism of controlling incorrect response [21]. However, a recent study provided evidence that fought against the opinion of N2 representing response-inhibition, and their results showed that N2 should represent conflict monitoring [23] and previous explanations of the N2 as indexing response inhibition might need to be revised. Moreover, other studies also suggested that the N2 might reflect conflict monitoring on correct response trials [24] and the high degree of response conflict might derive from the competition between the execution and the inhibition of a single response in go/no-go tasks [25]. In the current study, when participants confronted the choices of a beautiful female and money or a sexy female and money, they might have different degrees of response conflicts in the two tasks. The response time in task S was longer than in task B and the money amount of the cash equivlent in task S was generally higher than in task B. Thus, the cognitive response conflict (wanting both money and an attractive female) in task S might be larger than that in task B. Task S elicited a greater negativity (N2) than task B, might be related to larger conflict monitoring.

During the 550–1000 ms time window, choices in task S were accompanied by higher positive amplitudes (LPC) than those in task B. LPC was generally considered to relate to valuation processes [14], [16]. Different valuation processes may involve different subjective values. Mainly, there are two kinds of subjective values: decision value (the value that people assign to the options, and on which they base their decisions) and experienced value (the value that people experience when they get and consume something) [26]. According to the above definition and our experimental procedure, we regarded the value in the current consideration as decision value rather than experienced value. In our experiment, cash equivalents for sexy female images were generally higher than those for beautiful ones, indicating that sexy female images had higher decision value than beautiful ones, and so sexy images induced higher LPC than beautiful ones, as observed in the current experiment.

Another different but related explanation is as follows. Previous studies have found that P600 and other late positive ERP components were involved in erotic content in both males and females [27]–29. A recent study used unique design directly to compare the effects of erotic and non-erotic stimuli [12], and their results showed that erotic pictures yielded significantly higher positive slow waves (PSW) between 500 and 750 ms than sport stimuli with comparable arousal and valence levels. It was suggested that the larger arousal value and positive valence of erotic stimuli, both of which were evolutionarily relevant, were the core element of ‘motivated attention’ and played an important role during motivation-directed behaviour [16]. Thus, we inferred that evolutionary relevance might be a factor that accounted for our observed results. Specifically, sexy stimuli can be regarded as more relevant to survival and reproduction of human beings than beautiful stimuli [30], and so participants put higher subjective values to sexy females than beautiful females, and thus the former induced larger LPC than the latter.

In addition, during the 550–1000 ms time window, the positivity of choosing an attractive female picture was significantly larger than choosing a certain amount of money (cash equivalent). Imagine a male faced a vague image of an attractive female and needed to decide to view its clear version or to gain a fixed amount of money which was *roughly* as attractive as this kind of images. If he assigned relatively higher subjective decision value to this vague image, then (i) the corresponding ERP amplitude would be relatively larger and (ii) he would choose to view its clear version, because the decision value of this image was slightly higher than that of the fixed amount of money. If he assigned relatively lower subjective decision value to this vague image, then (i) the corresponding ERP amplitude would be relatively smaller and (ii) he would choose to gain a fixed amount of money, because the decision value of this image was slightly lower than that of the fixed amount of money. Therefore, choosing images of attractive females were preceded by larger amplitude of LPC, as observed in the current experiment.

Another possible explanation is, relative to the money amount, female pictures, no matter sexy ones or beautiful ones, were more directly related to humans' survival and reproduction, and their relevant cognitive and affective responses occurred earlier in the human beings' evolution. First, according to previous studies [12], [16], erotic stimuli were especially relevant to human's survival and reproduction. Second, a person with a more beautiful face was often supposed to be friendlier [31], and so a more beautiful face could be socially more rewarding. In addition, when making social decisions, humans might take the degree of beauty of faces into account. Both sexy and beautiful female images could begin to play an important role in human life in the early stage of evolution. Finally, compared with attractive females, money occurred quite late in the human evolutionary history, and the rewarding value of money was indirect (money itself cannot satisfy your appetite or any other kind of desire). In a word, as a kind of rewards, attractive female images were more direct and evolutionarily deeper than money, and so could induce stronger neural responses than their cash equivalents. When the subjects chose attractive female pictures over cash equivalent, they anticipated more direct rewards and so had stronger LPC.

In conclusion, the current study delineated the neural processes of the decision-making between attractive females and money in male subjects using ERP technique. Our findings clearly indicated that in the time window of 290–340 ms, task B evoked a larger P2 than task S, which may be related to a process of matching external perception with an internal representation or expectation in the early stage of the processing; in the time window of 340–390 ms, task S evoked a larger N2 than task B, which may relate to the response conflict and cognitive monitoring; finally, during the 550–1000 ms time window, decision-makings in task S were accompanied by significantly higher positive ERP components than those in task B, reflecting sexy female images may have higher decision value than beautiful ones, which may be because the former is evolutionarily more significant; more importantly, choosing an attractive female was accompanied by a larger late positive component than choosing money and this is possibly because the decision values of the chosen attractive female images were generally higher than those of the un-chosen ones, or because attractive female images are more direct and evolutionarily earlier rewards than money amounts, and this can influence the subjective evaluation and expectation process. Our study represents the first attempt to understand the neural processes of decisions between attractive females (a typical example of primary rewards) and money (a typical example of secondary rewards).
